# Supplementary material for: Rapid dynamics of dorsal raphe serotonin neurons regulate the strength of visual attention
Source: Nat Commun. 2026 Mar 18;17:3464. doi: 10.1038/s41467-026-70658-y (PMC13076638; doi:10.1038/s41467-026-70658-y)
Supplement: Supplementary file 1 — Supplementary Information [file 41467_2026_70658_MOESM1_ESM.pdf]

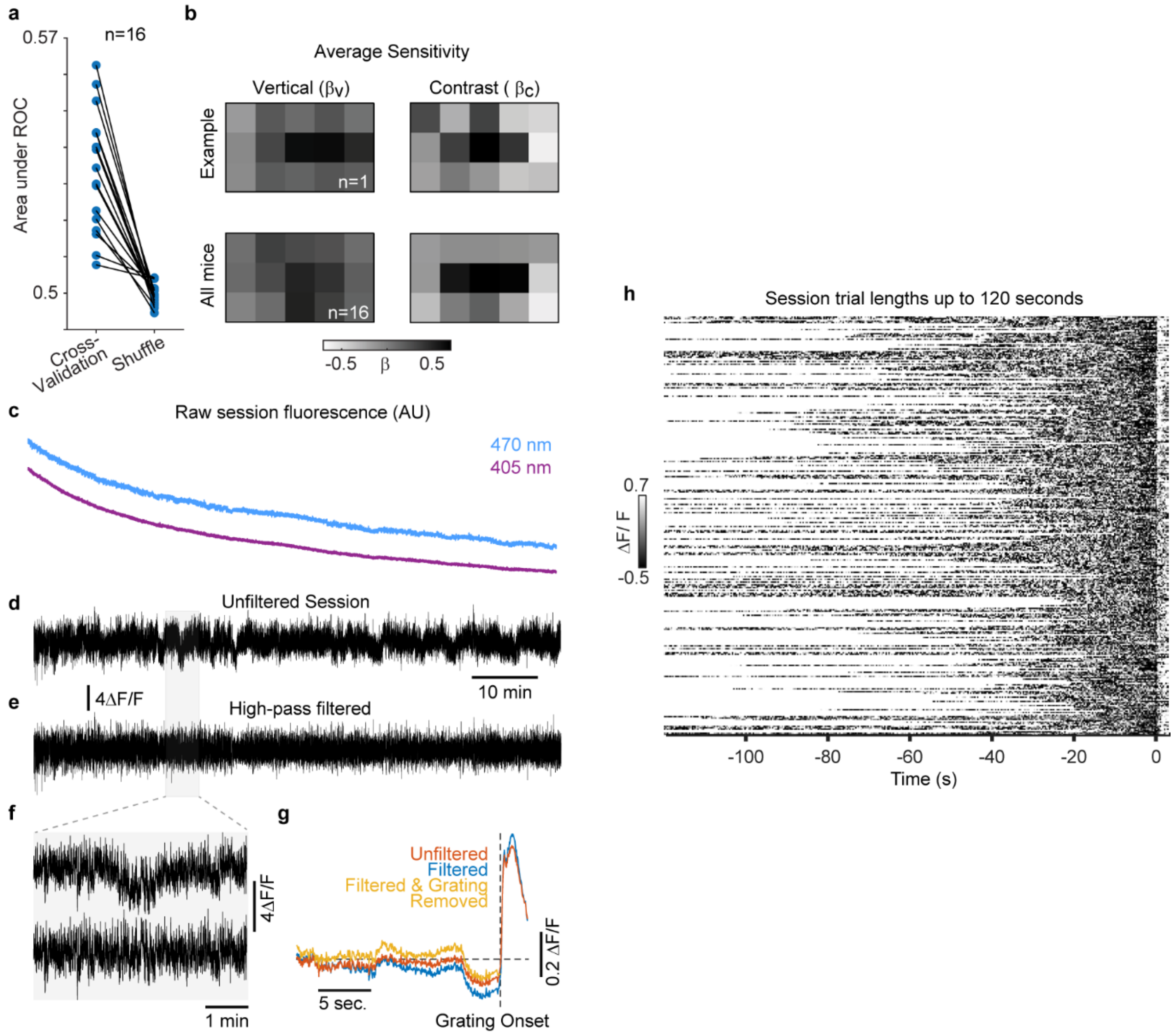

Supplementary Figure 1. Attention Model Spatial filters and Validation; Photometry processing a, Cross-validated area under the receiver operator characteristic (ROC) for 16 mice compared to model fits in which checkerboard sequences were shuffled relative to false alarm licks. **b,** Model sensitivity maps for vertical (βv) and contrast (βc) energy obtained from an example mouse (top) and average model sensitivity maps from 16 mice. **c,** Example session fluorescence signal from evoked by 470nm and 405nm LEDs during photometry. **d,** The same 470nm signal as in panel a following regression to the 405 signal. **e,** A high-pass filtered version of the signal in d. **f,** Magnified view of the gray boxed region of the unfiltered and filtered signals in d-e. **g,** Example photometry hit signals taken from the indicated processing stages illustrated in d-f and with grating removed (see methods). **h,** Raster shows example DR-5HT photometry signals from individual trials; 120 seconds are shown.

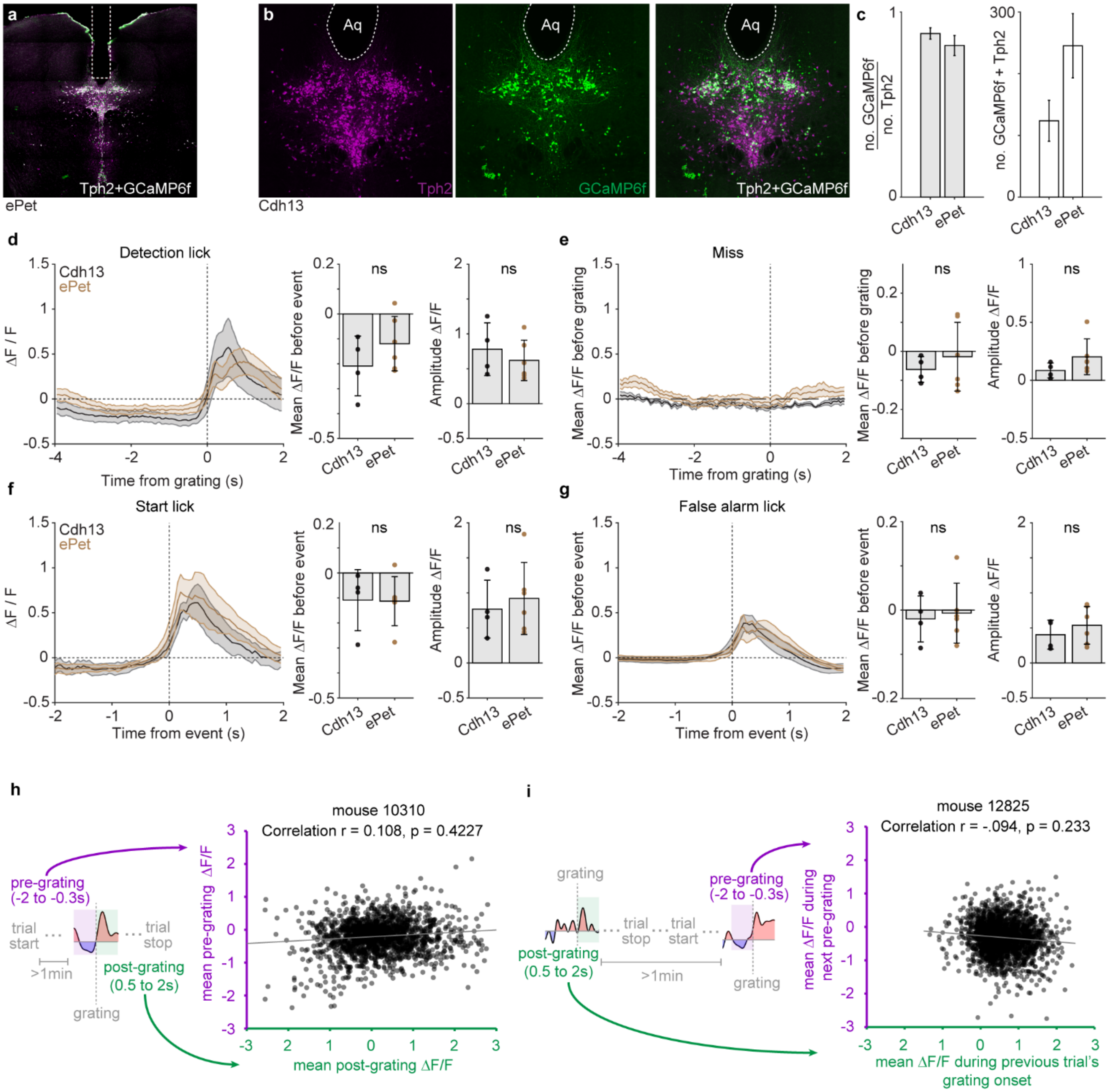

Supplementary Figure 2. Comparison of GCaMP6f signals from Cdh13-CreER and epet1-Cre mice and analysis of spatial sensitivities to contrast and vertical energy during high and low DR-5HT activity a, Example coronal section through the DR of an epet1-Cre x Cre-dependent GCaMP6f (Ai95) mouse stained with antibodies against tryptophan hydroxylase 2 (Tph2) and GFP. **b,** Example coronal section taken from a Cdh13-CreER x Ai95 mouse stained for Tph2 (left) and GFP (middle). Right image shows the merge. **c,** Fraction (left) and absolute number of Tph2+ neurons that are also GCaMP6f+ in epet1-Cre x Ai95 and Cdh13-CreER x Ai95 mice. Both lines label similar numbers of Tph2+ neurons with GCaMP6f but epet1-Cre produces more complete labelling (n=3 epet1-Cre mice and n=3 Cdh13-CreER mice). **d-g,** Average GCaMP6f photometry signals from epet1-Cre or Cdh13-CreER mice aligned to detection (d), miss (e), start (f), and False alarms (g). Bar graphs at right quantify the mean ΔF/F prior to the indicated event and the mean post-event amplitude. **h,** Left: example DR activity during pre- and post-grating DR activity for one hit trial. Right: no correlation between pre- and post-grating DR activity for hit trials. **i,** Left: example photometry showing DR activity during grating and during the subsequent trial's pre-grating period. Right: no correlation between grating ΔF/F and the subsequent trial's pre-grating ΔF/F for hit trials. Pre- and post-grating intervals indicated.

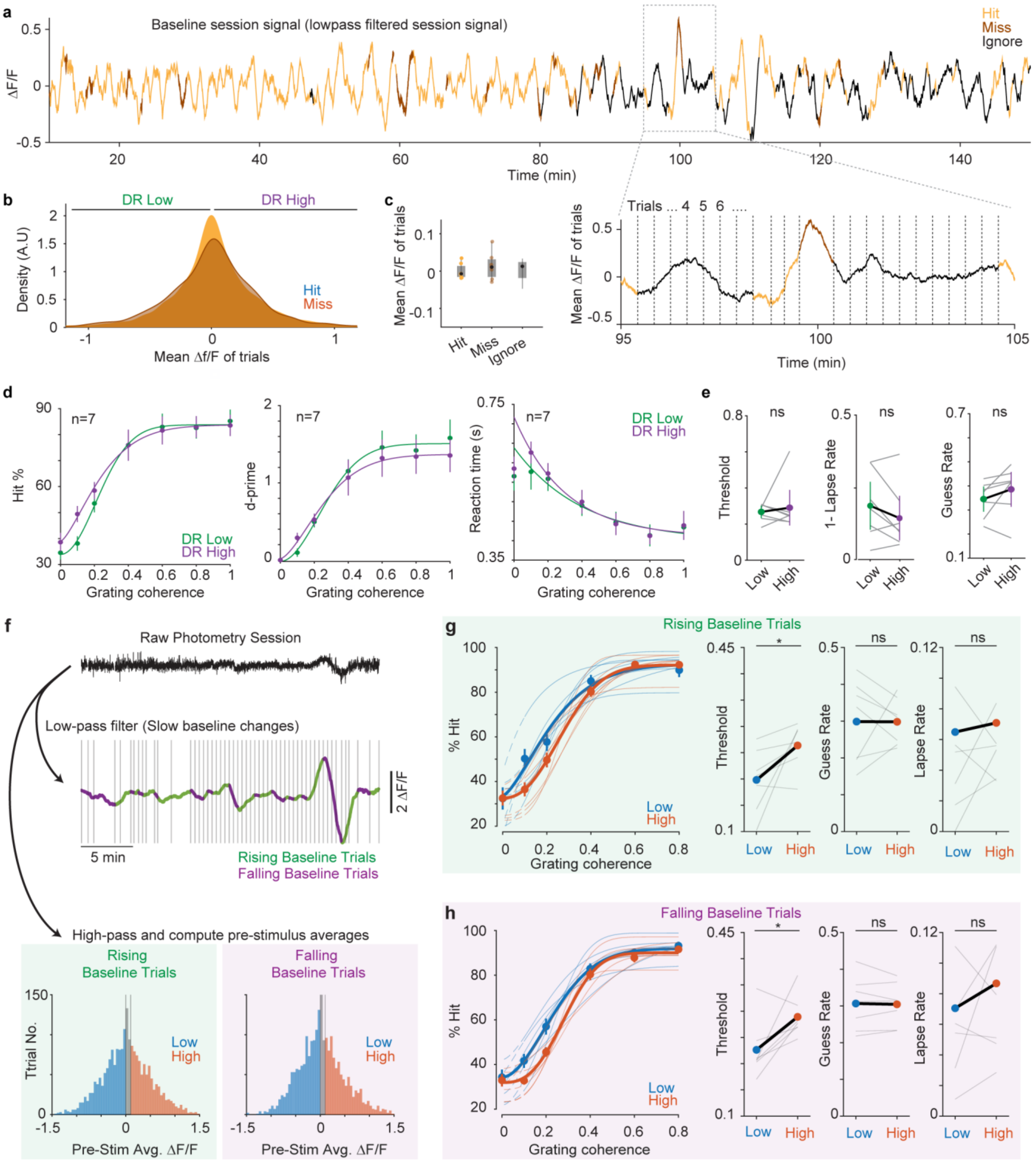

Supplementary Figure 3. Long-timescale DR dynamics and behavioral performance. a, Example trace showing changes in DR baseline activity across ~150 minutes. Trace is colored according to hit, miss and ignore trials. Inset magnifies the boxed region. **b,** Histogram of baseline ΔF/F during hits and misses taken from session signals like shown in a. **c,** Average ΔF/F for hit, miss, and ignore trials. **d,** Psychometric curves of hit rate, d-prime, and reaction time versus grating coherence **e,** Average psychometric threshold (left), lapse rate (middle) and FA rate (right) computed from data like shown in d. n> 1500 baseline segments from 7 mice. **f,** Raw photometry signals from an entire session low pass filtered to identify rising and falling baseline periods (middle) and parallel high-pass filtering to identify fast DR-high and low signals (bottom). **g,** Mean psychometric curves of hits and corresponding measures of psychometric threshold, guess rate and lapse rate for DR-high and low trials occurring on a rising baseline. **h,** Mean psychometric curves of hits and corresponding measures of psychometric threshold, guess rate and lapse rate for DR-high and low trials occurring on a rising baseline. Slow changes in baseline are not related to detection and behavioral performance (n=7 mice).

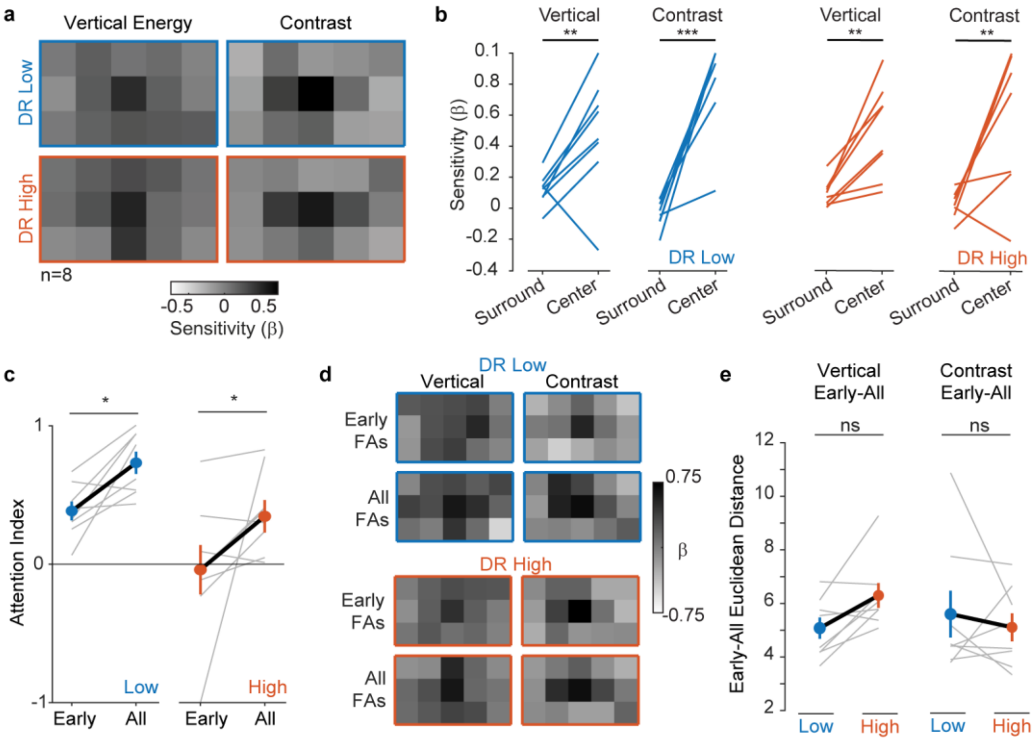

Supplementary Figure 4. Spatial sensitivities to visual features are unaffected by DR activity and block switches. a, Average model sensitivity maps for vertical and contrast energy computed from false alarms (FAs) preceded by high or low DR-5HT activity. **b,** Vertical and contrast sensitivities averaged for the central and all surrounding checker bins for 8 individual mice during DR-5HT low and DR-5HT high. Sensitivity remains at the central bin regardless of changes in DR-5HT activity prior to FAs. **c,** Average attention indices computed from low or high DR-5HT signals preceding false alarms (FAs) occurring either early in a block (first 5-15 false alarms in block) or to all FAs (n=6). Attention index grows across trials within a block. **d,** Spatial sensitivities (βc and βv) computed from FAs occurring either early in a block or to all FAs and preceded by either high or low DR-5HT activity. **e,** Quantification of the sensitivity maps shown in d.

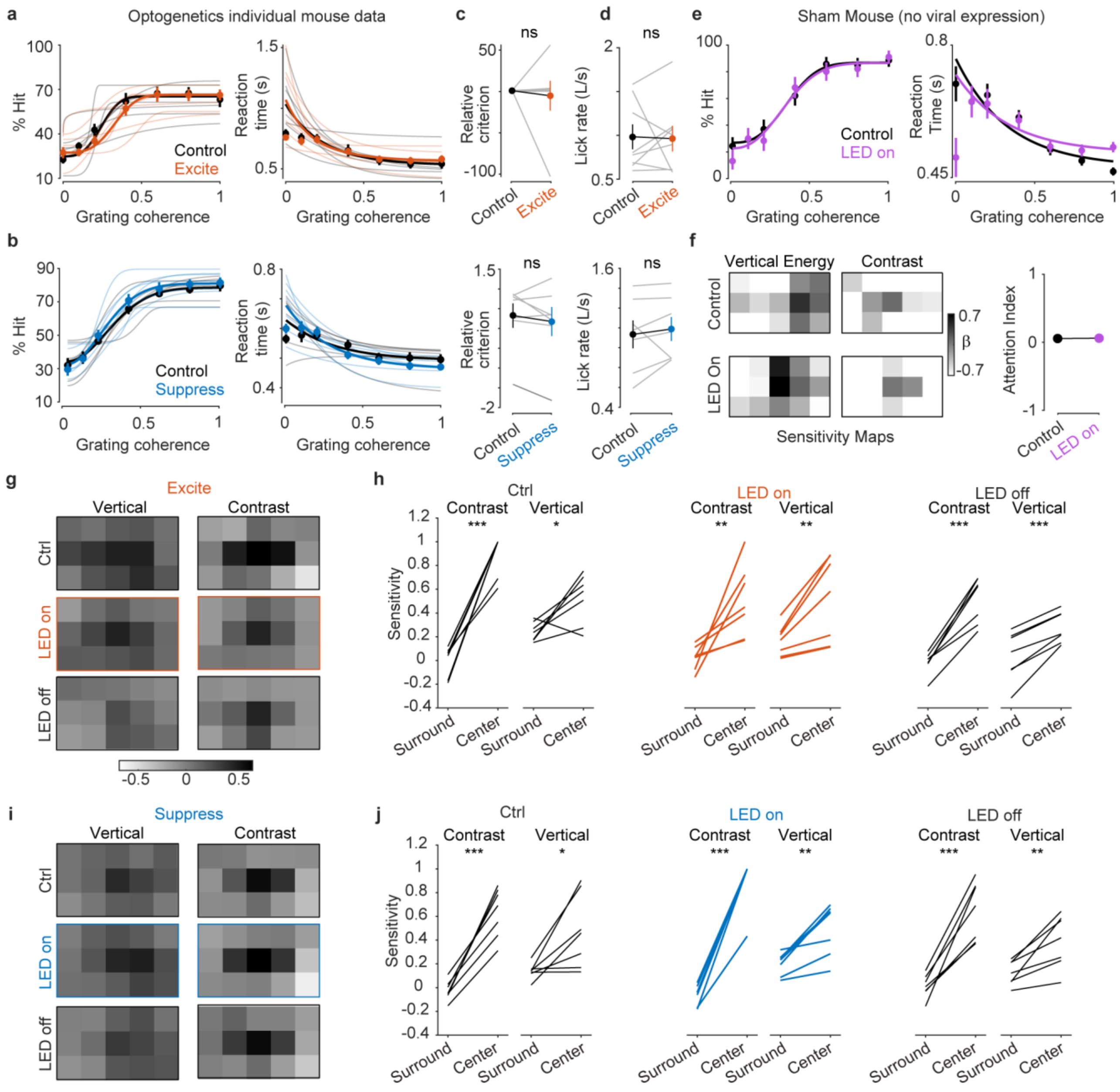

Supplementary Figure 5. Mice for optogenetic experiments were trained to similar performance level as controls. a-b, % hits (left) and reaction time (right) versus grating coherence computed from control trials or trials with optogenetic excitation (a) and suppression (b). **c-d,** Relative criterion (c) and mean lick rate (d) computed from experiments like those in a-b and in Figure 4e and j. **e,** Results of optogenetic experiment in a sham mouse that received the same fiber optic implant but did not express ChR2 or Jaws. % hits (left) and reaction time (right) versus grating coherence computed from control trials or trials with optical excitation in a sham mouse. **f,** Model sensitivity maps (left) and attention (right) for the sham experiment shown in e. **g,** Average model sensitivity maps for vertical and contrast energy computed from false alarms (FAs) before optogenetic stimulation (ctrl), FAs preceded by optical stimulation (LED On), and FAs not preceded by stimulation. **h,** Vertical and contrast sensitivities averaged for the central and all surrounding checker bins for individual mice, like those shown in g. **i,** Average model sensitivity maps for vertical and contrast energy computed from false alarms (FAs) before optogenetic suppression (ctrl), FAs preceded by optical suppression (LED On), and FAs not preceded by suppression. **j,** Vertical and contrast sensitivities averaged for the central and all surrounding checker bins for individual mice, like those shown in i.

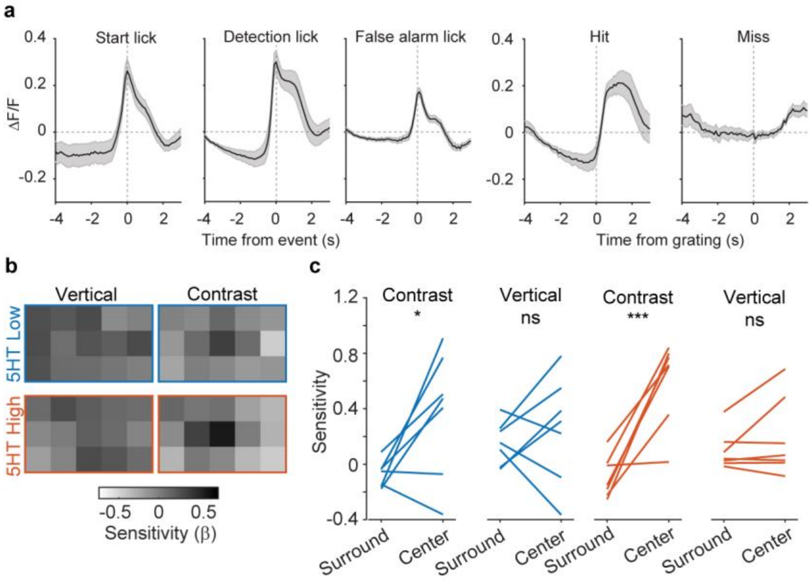

Supplementary Figure 6. GRAB5HT signals from V1 aligned to task events and outcomes. a, V1 GRAB5HT signals aligned to start, detection, and false alarm licks (task events), and to hits and misses (task outcomes). **b,** Average model sensitivity maps for vertical and contrast energy computed from false alarms (FAs) preceded by high or low GRAB5HT signals. **c,** Vertical and contrast sensitivities averaged for the central and all surrounding checker bins for individual mice during 5HT low and 5HT high.
